# Thiadiazole derivatives as anticancer agents

**DOI:** 10.1007/s43440-020-00154-7

**Published:** 2020-09-03

**Authors:** Monika Szeliga

**Affiliations:** Department of Neurotoxicology, Mossakowski Medical Research Centre, Polish Academy of Sciences, 5 Pawinskiego Str, 02-106 Warsaw, Poland

**Keywords:** Thiadiazole derivatives, Cancer, Anticancer therapy, Clinical trials

## Abstract

In spite of substantial progress made toward understanding cancer pathogenesis, this disease remains one of the leading causes of mortality. Thus, there is an urgent need to develop novel, more effective anticancer therapeutics. Thiadiazole ring is a versatile scaffold widely studied in medicinal chemistry. Mesoionic character of this ring allows thiadiazole-containing compounds to cross cellular membrane and interact strongly with biological targets. Consequently, these compounds exert a broad spectrum of biological activities. This review presents the current state of knowledge on thiadiazole derivatives that demonstrate in vitro and/or in vivo efficacy across the cancer models with an emphasis on targets of action. The influence of the substituent on the compounds’ activity is depicted. Furthermore, the results from clinical trials assessing thiadiazole-containing drugs in cancer patients are summarized.

## Introduction

According to the most recent data provided by the International Agency for Research on Cancer (IARC), 18.1 million new cases and 9.6 million cancer deaths were registered worldwide in 2018 [1]. Due to the population aging and growth, the number of new cancer cases is expected to increase. Although a substantial progress was made in the understanding of molecular biology of particular cancer types, and numerous potential specific therapeutic targets were identified in recent years, there is an urgent necessity for the development of improved anticancer therapeutic strategies.

**Fig. 1:**
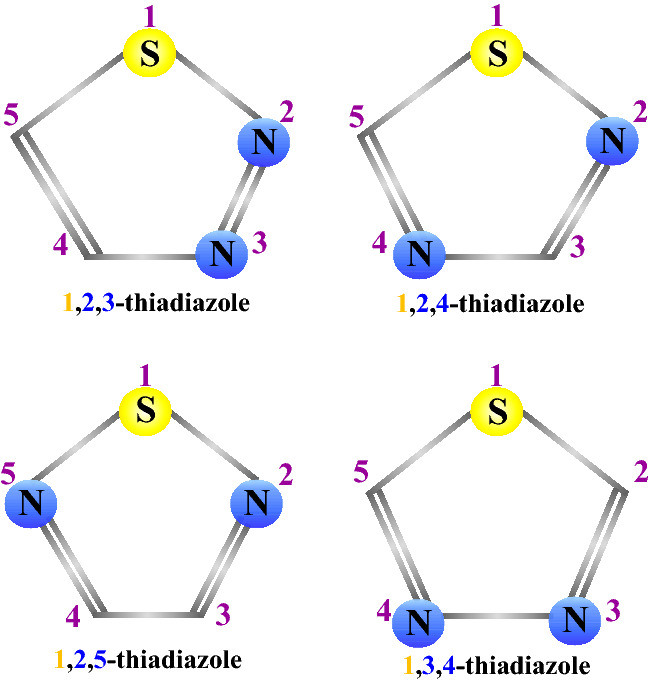
Core structures of the thiadiazole isoforms occurring in nature. Sulphur and nitrogen atoms are marked as yellow or blue circles, respectively

Thiadiazole is a five-membered heterocyclic compound containing one sulfur and two nitrogen atoms. It occurs in nature in four isoforms: 1,2,3-thiadiazole, 1,2,4-thiadizaole, 1,2,5-thiadiazole and 1,3,4-thiadiazole (Fig. 1). Taking into account that thiadiazole is the bioisostere of pyrimidine and oxadiazole, it is not surprising that compounds bearing this moiety present a broad spectrum of pharmacological properties, including antiviral, antibacterial, antifungal, antiparasitic, anti-inflammatory and anticancer activities [2]. Due to the mesoionic nature, thiadiazoles are able to cross the cellular membranes. Their relatively good liposolubility is most likely attributed to the presence of the sulphur atom [3]. The thiadiazole-containing drugs, including diuretics acetazolamide and methazolamide or antibiotics cefazedone and cefazolin sodium, are already used in clinics. Accumulating evidence has also revealed numerous thiadiazole derivatives that display anticancer activities in various in vitro and in vivo models (summarized in Table 1). Moreover, several thiadiazole-containing compounds have moved into clinical trials either as single agents or in combination with existing anticancer drugs (summarized in Table 2).

**Table 1:** Summary of the anticancer activities of the thiadiazole derivatives in vitro and in vivo

Class of compounds	Target	Outcome	References
**Derivatives of 1,2,3-thiadiazole**			
Analogs of combretastatin A-4 (CA-4) containing 1,2,3-thiadiazole	Tubulin polymerization	Decreased proliferation of human myeloid leukemia HL-60, colon adenocarcinoma HCT-116, immortalized human microvascular endothelial HMEC-1 cells; reduced tumor growth in mice S180 sarcoma model	[5]
5-Aryl-4-(5-substituted-2-4-dihydroxyphenyl)-1,2,3-thiadiazoles	Hsp90	Decreased viability of human cervical carcinoma HeLa and osteosarcoma U2OS cells	[7]
		Decreased proliferation of colon adenocarcinoma HCT-116 cells; induction of apoptosis	[8]
d-ring fused 1,2,3-thiadiazole dehydroepiandrosterone (DHEA) derivatives	Unknown	Decreased proliferation, ability to form colonies and migrate of human breast cancer T47D cells; induction of apoptosis	[9]
		Reduced tumor growth and metastatic ability in T47D xenografts	[10]
Pyrazole oxime derivatives bearing 1,2,3-thiadiazole	Unknown	Decreased viability of human hepatocarcinoma Huh-7, pancreatic Panc-1, colon HCT-116, gastric SGC-7901 cancer cells	[11]
**Derivatives of 1,2,4-thiadiazole**			
3,5-Dipyridyl-1,2,4-thiadiazoles	Unknown	Decreased proliferation of human breast cancer MCF-7 cells	[13]
3-Substituted benzo[4,5]imidazo[1,2-d] [1,2,4]thiadiazole	Unknown	Decreased viability of human myeloid leukemia HL-60, U937 and melanoma SK-MEL-1 cells; induction of apoptosis	[14]
**Derivatives of 1,2,5-thiadiazole**			
Anthra[2,1-c] [1,2,5]thiadiazole-6,11-dione (NSC745885)	IKKβ	Decreased proliferation of human leukemia, melanoma, ovarian, breast, prostate cancer and glioma cells	[15, 19]
		Decreased viability of human oral cancer SAS cells; unchanged viability of human lung fibroblasts; reduced tumor growth of SAS xenografts; induction of apoptosis	[16]
		Decreased viability of human bladder cancer T24 and MBT2 cells, unchanged viability of immortalized normal urothelial cells SV-HUC-1 or normal fibroblasts 3T3	[18]
4-Chloroanthra[2,1-c] [1,2,5]thiadiazole-6,11-dione (NSC757963)	Unknown	Decreased viability of leukemia, breast, ovarian, prostate cancer, melanoma cells	[19]
4-(Isopropylthio)anthrax [1,2-c][1,2,5]thiadiazole-6,11-dione (NSC763968)	Unknown	Decreased viability of human leukemia, prostate, ovarian, breast, renal, colon cancer, melanoma, glioma, non-small cell lung cancer; relatively not toxic towards human urothelial SV-HUC-1 and prostate epithelial RWPE-1 cells; induction of apoptosis in prostate cancer DU-145 cells	[20]
Nitrogen-substituted anthra[1,2-c] [1,2,5] thiadiazole-6,11-dione (RV-59)	Unknown	Decreased viability of human colon cancer HCT-116 cells with high level of cytoplasmic Nrf2 (cNrf2); reduced growth of xenograft induced by HCT-116 cells with high cNrf2	[25]
**Derivatives of 1,3,4-thiadiazole**			
2-Ethylamino-1,3,4-thiadiazole (EATDA)	Unknown	Reduced growth of mammary adenocarcinomas in mice	[26]
2-Amino-1,3,4-thiadiazole (ATDA) (NSC 4728)	IMPDH	Reduced tumor growth in mice surviving systemic leukemia; prolonged survival time of the animals	[27]
1,3,4-Thiadiazole-2-sulfonamide derivatives	CA II, CA IV	Decreased viability of human leukemia, non-small cell lung cancer, ovarian, melanoma, colon, glioma, renal, prostate and breast cancer cells	[39]
Biphenyl-disulfonamide derivative bearing 5-amino-1,3,4-thiadiazole-2-sulfonamide	CA II, CA IX	Decreased viability of human colon cancer HCT-116 cells	[40]
Bis-2-(5-phenylacetamido-1,3,4-thiadiazol-2-yl)ethyl sulfide (BPTES)	GA	Decreased proliferation of human Burkitt lymphoma P493 cells; reduced lymphoma xenograft growth	[42]
		Decreased viability of glioblastoma, non-small cell lung cancer and acute myeloid leukemia cells	[43–45]
		Reduced hepatocellular carcinoma xenograft growth	[46]
*N*-(5-{2-[2-(5-Amino-[1,3,4]thiadiazol-2-yl)-ethylsulfanyl]-ethyl}-[1,3,4]thiadiazol-2-yl)-2-phenyl-acetamide	GA	Decreased viability of human Burkitt lymphoma P493 cells; reduced lymphoma xenograft growth	[48]
*N*-[5-[4-[6-[[2-[3-(trifluoromethoxy) phenyl]acetyl]amino]-3-pyridazinyl]butyl]-1,3,4-thiadiazol-2-yl]-2-pyridineacetamide (CB-839)	GA	Decreased proliferation of the triple negative breast cancer HCC1806 and MDA-MB-231 cells; reduced growth of breast cancer xenografts; no overt signs of toxicity	[49]
		Decreased viability of human *EGFR* mutant non-small cell lung cancer HCC827 cells; reduced xenograft growth	[50]
		Decreased colony formation of human lung cancer A427, A549 and H460 cells; reduced H460 xenograft tumor growth when combined with radiation	[51]
		Decreased viability of *NF1* mutant/null malignant peripheral nerve sheath tumor (MPNST) ST8814 and S462 cells; reduced growth of MPNST xenografts	[52]
		Reduced growth of undifferentiated pleomorphic sarcoma (UPS) xenografts	[54]
2-[5-(4-Substitutedphenyl)-[1,3,4]-thiadiazol-2-ylamino]-pyrimidine-5-carboxylic acid hydroxyamides	HDAC	Decreased viability of human colorectal carcinoma HCT-116 cells; reduced growth of tumors formed by Ehrlich ascites carcinoma (EAC) cells	[58]
Amino-1,3,4-thiadiazole-based hydroxamic acid derivatives	HDAC	Decreased viability of human breast MDA-MB-231, prostate PC3 cancer and chronic myelogenous leukemia K562 cells	[59, 60]
5-Substitutedphenyl-1,3,4-thiadiazole-based hydroxamic acids	HDAC	Decreased viability of human colon cancer SW620, breast cancer MCF7, prostate cancer PC3, pancreas cancer AsPC1 and lung cancer NCI-H460 cells	[61]
*N*-(4-Acetyl-4,5-dihydro-5-methyl-5-phenyl-1,3,4-thiadiazol-2-yl)acetamide (K858)	Eg5	Decreased viability of human colon cancer HCT-116 cells; reduced tumor growth of human A2780 ovarian cancer xenografts; no overt evidence of toxicity	[63]
		Decreased viability of human prostate PC3, breast MCF7, MDA-MB231, BT474, SKBR3 cancer, melanoma SK-MEL-5, SK-MEL-28 and glioblastoma U87, U251 cells	[64–66]
(2*S*)-2-(3-Aminopropyl)-5-(2,5-difluorophenyl)-*N*-methoxy-*N*-methyl-2-phenyl-1,3,4-thiadiazole-3(2*H*)-carboxamide trifluoroacetate (Filanesib, ARRY-520)	Eg5	Decreased viability of leukemic U937, Jurkat, and HL-60, Molm13 and OCI-AML3 cells; reduced tumor growth of HL-60 xenografts	[67]
		Decreased viability of colon HT-29, breast UISO-BCA-1, prostate PC3 cancer, myeloma RPMI8226, JJN3, U266, and NCI H929 cells; reduced growth of xenografts formed by above mentioned cells	[68, 69]
*N*-[(5*R*)-4-(2,2-dimethylpropanoyl)-5-[[2(ethylamino) ethylsulfonylamino] methyl]-5-phenyl-1,3,4-thiadiazol-2-yl]-2,2-dimethylpropanamide (Litronesib, LY2523355)	Eg5	Decreased viability of human colon cancer HCT-116 cells; induction of apoptosis; reduced growth of patient-derived xenografts	[74]
Imidazo[2,1‐*b*] [1,3,4]thiadiazolindolin‐2‐ones	Tubulin polymerization	Decreased viability of human lung A549, cervical HeLa, breast MCF‐7, and colon HCT-116 cancer cells	[77, 78]
5-[(4-Fluorobenzoyl)amino]-2-[(4-fluorobenzyl)thio]-1,3,4-thiadiazole	Abl	Decreased proliferation and increased differentiation of human leukemia HL-60 cells	[81]
*N*-(5-Nitrothiazol-2-yl)-2-((5-((4-(trifluoromethyl)phenyl)amino)-1,3,4-thiadiazol-2-yl)thio)acetamide	Abl	Decreased viability of human leukemia K562, MT-2, Jurkat and cervical carcinoma HeLa cells	[82]
Compounds bearing 1,3,4-thiadiazole and phthalimide residues	LOX	Decreased viability of colon adenocarcinoma HT29 and neuroblastoma SKNMC cells	[84]
*N*-(5-(ppyridin-2-yl)-1,3,4-thiadiazol-2-yl)benzamide derivatives	LOX	Decreased viability of prostate cancer PC3, colon adenocarcinoma HT29 and neuroblastoma SKNMC cells	[85]
({(3-(5-Amino-1,3,4-thiadiazol-2-yl)-1-cyclopropyl-6-fluoro-7-(piperazin-1-yl)quinolin-4(1*H*)-one)})	DNA	Decreased viability of hepatocellular carcinoma Huh-7 cells	[87]
Hybrids of 1,3,4-thiadiazole and chalcone containing phenolic moiety	DNA	Decreased viability of leukemia HL-60, cervical cancer HeLa, lung carcinoma A549 cells and normal lung MRC-5 cells	[88]
2-(4-Bromophenylamino)-5-(2,4-dichlorophenyl)-1,3,4-thiadiazole	TopoII	Decreased viability of breast cancer MCF-7 and MDA-MB-231 cells; unaffected viability of normal fibroblasts	[90]
5-Substituted 2(2,4-dihydroxyphenyl)-1,3,4-thiadiazoles	Unknown	Decreased viability of bladder HCV29T, breast T47D cancer, non-small lung carcinoma A549 and rectal adenocarcinoma SW707 cells	[91]
*N*-Aryl ring-substituted 2-phenyloamino-5-(2,4-dihydroxyphenyl)-1,3,4-thiadiazoles	Unknown	Decreased viability of bladder HCV29T, breast T47D cancer, non-small lung carcinoma A549 and rectal adenocarcinoma SW707 cells	[93]
*N*-Substituted 2-amino-5-(2,4-dihydroxyphenyl)-1,3,4-thiadiazoles	Unknown	Decreased viability of bladder HCV29T, breast T47D cancer, non-small lung carcinoma A549 and rectal adenocarcinoma SW707 cells	[94]
2-(4-Chlorophenylamino)-5-(2,4-dihydroxyphenyl)-1,3,4-thiadiazole (CPDT)	Unknown	Decreased viability of human breast T47D, thyroid FTC238, colon HT-29 carcinoma, leukemia Jurkat, medulloblastoma TE671, astrocytoma MOGGCCM, mouse teratoma P19 and rat glioma C6 cells; unaffected viability of rat astrocytes, neurons, hepatocytes and human fibroblasts	[95]
2-(4-Fluorophenyloamino)-5-(2,4-dihydroxyphenyl)-1,3,4-thiadiazole (FPDT)	Unknown	Decreased viability of human colon cancer HT-29, lung carcinoma A549, medulloblastoma TE671, human neuroblastoma SK-N-AS and rat glioma C6 cells; unaffected viability of rat astrocytes, neurons and hepatocytes	[96]
3-(Imidazo [2,1-b] [1, 3, 4]thiadiazol-2-yl)-1*H* indole analogues	Unknown	Decreased viability of pancreatic SUIT-2, Capan-1 and Panc-1 cells	[100, 101]

*Hsp90* heat shock protein 90, *IKKb* inhibitor of nuclear factor kappa-B kinase subunit beta, *IMPDH* inosine monophosphate dehydrogenase, *CA* carbonic anhydrase, *GA* glutaminase, *HDAC* histone deacetylase, *Eg5* kinesin spindle protein, *Abl* Abl kinase, *LOX* lipoxygenase, *TopoII* topoisomerase II

**Table 2:** Thiadiazole derivatives in clinical trials

Agent	Disease	Clinical trial number/references	Status/outcome
**Inhibitors of inosine monophosphate dehydrogenase (IMPDH)**			
NSC 4728	Solid tumors	Phase I/[30]	Completed; PR: 5%
	Non-small cell lung carcinoma	Phase II/[30]	Completed; PR: 7%
	Renal cell carcinoma	Phase II/[31]	Completed; PR: 2%
	Colon cancer	Phase II/[32]	Completed; PR: 12%
	Squamous carcinoma of the cervix	Phase II/[33]	Completed; PR: 5%; SD: 28%
	Non-squamous cervical carcinoma	Phase II/[34]	Completed; CR: 8%; SD: 35%
	Mixed mesodermal tumors of the uterine corpus	Phase II/[35]	Completed; PR: 5%
	Leiomyosarcoma	Phase II/[36]	Completed; lack of clinical activity
	Epidermoid carcinoma of the esophagus	Phase II/[37]	
**Inhibitors of carbonic anhydrase (CA)**			
Acetazolamide + platinium + etoposide-based radiochemotherapy	Small cell lung cancer	NCT03467360 phase I	Completed
Acetazolamide + temozolomide	Malignant glioma	NCT03011671 phase I	
**Inhibitors of glutaminase (GA)**			
CB-839	Leukemia	NCT02071927 phase I	Completed
	Hematologic malignancies	NCT02071888 phase I	
	Solid tumors	NCT02071862 phase I	
CB-839 + paclitaxel or panitumumab	Triple negative breast cancer	NCT03057600 phase II	
CB-839 + nivolumab	Clear cell renal cell carcinoma, melanoma, and non-small cell lung cancer	NCT02771626 phase I/II	Active
CB-839 + everolimus	Renal cell carcinoma	NCT03163667 phase II	
CB-839 + niraparib	Ovarian cancer, *BRCA*^wt^	NCT03944902 phase I	
CB-839 + cabozantinib	Renal cell carcinoma	NCT03428217 phase II	
CB-839 + talazoparib	Solid tumors	NCT03875313 phase I/II	Recruiting
CB-839 + capecitabine	Solid tumors	NCT02861300 phase I/II	
CB-839 + azacitidine	Myelodysplastic syndrome	NCT03047993 phase I/II	
CB-839 + radiation therapy + temozolomide	*IDH*^mut^ diffuse or anaplastic astrocytoma	NCT03528642 phase I	
CB-839 + osimertinib	Non-small cell lung cancer, *EGFR*^mut^	NCT03831932 phase I/II	
CB-839	Solid tumors	NCT03872427 phase II	
CB-839 + carfilzomib + dexamethasone	Multiple myeloma	NCT03798678 phase I	
CB-839 + palbociclib	Solid tumors	NCT03965845 phase I/II	
CB-839 + sapanisertib	Non-small cell lung cancer	NCT04250545 phase I	Not yet recruiting
CB-839 + pembrolizumab	Non-squamous, non-small cell lung cancer	NCT04265534 phase II	
**Inhibitor of kinesin spindle protein (KSP, Eg5)**			
Filanesib (ARRY-520)	Advanced myeloid leukemia	NCT00637052 phase I/[70]	Completed; lack of clinical activity
	Multiple myeloma	NCT02092922 phase II	Completed
	Advanced solid tumor	NCT00462358 phase I/[71]	Completed; lack of clinical activity
Filanesib + bortezomib + dexamethasone	Multiple myeloma	NCT01248923 phase I/[72]	Completed; ORR: 20%; CBR: 33%; DCR: 65%
Filanesib alone + dexamethasone	Multiple myeloma	NCT00821249 phase I/II/[73]	Completed; ORR: 16%; CBR: 23% ORR: 15%; CBR: 20%
Filanesib + carfilzomib	Multiple myeloma; plasma cell leukemia	NCT01372540 phase I	Completed
	Multiple myeloma	NCT01989325 phase II	
Filanesib + pomalidomide + dexamethasone	Multiple myeloma	NCT02384083 phase I/II	
Litronesib	Solid tumors	NCT01358019 phase I/[75]	Completed lack of clinical activity
Litronesib alone or plus pegfilgrastim	Advanced solid tumors	NCT01214629 phase I NCT01214642 phase I/[76]	Completed; PR: 2%; SD: 20%
Litronesib + pegfilgrastim	Metastatic breast cancer	NCT01416389 phase II	Completed
	Small cell lung cancer	NCT01025284 phase II	
	Ovarian, non-small cell lung, prostate, colorectal, gastroesophageal cancers; squamous cell carcinoma of the head and neck	NCT01059643 phase II	

*CR* complete response, *PR* partial response, *SD* stable disease, *ORR* overall response rate; *CBR* clinical benefit rate, *DCR* disease control rate

## Derivatives of 1,2,3-thiadiazole

### Inhibitors of tubulin polymerization

Microtubules are cytoskeleton filamentous proteins built by tubulin. They are involved in numerous cellular processes such as intracellular transport, cell signaling, mitosis, cellular integrity and gene expression, but also contribute to polarity and shape of cells. A growing body of evidence documents anticancer activity of different heterocyclic compounds inhibiting tubulin polymerization [4].

Wu and co-workers focused on analogs of the *cis* stilbene derivative combretastatin A-4 (CA-4), an anticancer agent which binds to tubulin and inhibits microtubule polymerization. *Cis* configuration of the double bond in olefin group and 3,4,5-trimethoxyphenyl group are crucial for the CA-4’s activity. The newly designed and synthetized analogs contained 1,2,3-thiadiazole instead of the CA-4’s olefin group. They exhibited a diverse cytotoxicity against human myeloid leukemia HL-60 cell line, human colon adenocarcinoma HCT-116 cell line, and immortalized human microvascular endothelial (HMEC-1) cells. In all three cell lines, several tested compounds presented cytotoxic activity similar to that of CA-4 or lower, but still considerable (IC_50_ ranging from 13.4 to 86.6 nM). Of note, if the 3,4,5-trimethoxyphenyl was at 4th position in 1,2,3-thiadiazole, six out of nine tested compounds displayed significant activity in all three cell lines, while if this substituent was at 5th position, only one out of nine compounds was cytotoxic. These compounds inhibited tubulin polymerization with activities quantitatively similar to those of CA-4 and arrested the cell cycle at G2/M phase. Two of these derivatives significantly reduced tumor growth in mice S180 sarcoma model with the inhibition rate comparable or even higher to that of CA-4 [5].

### Inhibitors of Hsp90

The other group of 1,2,3-thiadiazole derivatives appeared to block the activity of heat shock protein 90 (Hsp90). Hsp90 displays a chaperone activity and controls the folding of numerous proteins. Inhibition of its activity results in the degradation of several oncoproteins. A growing body of evidence shows that tumor cells are more susceptible to blocking of Hsp90 compared to normal cells, therefore, this protein seems to be a promising anticancer target [6].

**Fig. 2:**
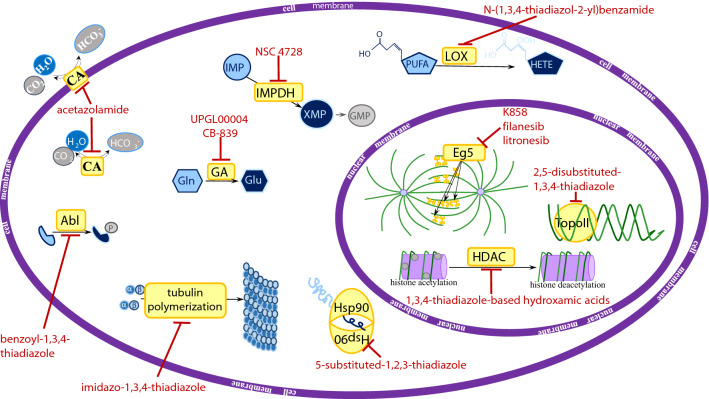
The molecular targets of the thiadiazole derivatives. Thiadiazole derivatives are shown in red. Molecular targets (yellow boxes): *CA* carbonic anhydrase, *Abl* Abl kinase; *GA* glutaminase, *IMPDH* inosine monophosphate dehydrogenase, *Hsp90* heat shock protein 90, *LOX* lipoxygenase, *Eg5* kinesin spindle protein, *HDAC* histone deacetylase, *TopoII* topoisomerase II. The other molecules: *Gln* glutamine, *Glu* glutamate, *IMP* inosine monophosphate, *XMP* xanthosine monophosphate, *GMP* guanosine monophosphate, *PUFA* polyunsaturated fatty acid, *HETE* hydroxyeicosatetraenoic acid

Cikotine et al. synthesized a series of 5-aryl-4-(5-substituted-2-4-dihydroxyphenyl)-1,2,3-thiadiazoles bearing at the position 5 of the thiadiazole ring one of the following groups: 4-MeOC_6_H_4_, 4-EtOC_6_H_4_, 4-MeC_6_H_4_ or 3,4-di-MeOC_6_H_3_ and either chloro- or ethyl- substituent at the position 5 of the dihydroxyphenyl moiety. Each of these derivatives tightly bound to Hsp90 (Fig. 2), and the strongest binding (dissociation constant (Kd) of 4.8 nM) displayed compound 3b, bearing a 4-EtOC_6_H_4_ substituent at position 5 of thiadiazole and a chlorine atom at position 5 of dihydroxyphenyl. Each of the derivatives significantly inhibited viability of both human cervical carcinoma HeLa and osteosarcoma U2OS cells and the most potent inhibitor appeared to be compound 3e bearing 4-EtOC_6_H_4_ group at the position 5 of the thiadiazole ring and an ethyl substituent at the position 5 of dihydroxyphenyl. The GI_50_ values of this compound were 0.70 μM for HeLa and 0.69 μM for U2OS cells, respectively. Of note, the other group of 5-aryl-4-(5-substituted-2-4-dihydroxyphenyl)-1,2,3-thiadiazoles bearing chloro-substituent at position 3 of the dihydroxyphenyl moiety did not bind to Hsp90 and was a very weak inhibitor of cancer cell viability. Most likely, the presence of this chloro-substituent prevented the formation of the extensive H-bonding network which in turn led to a lack of activity [7].

Three of the compounds, confirmed to bind Hsp90 most effectively, were subjected to further analysis. They exhibited antiproliferative activity against human colon cancer HCT-116 cells with GI_50_ values ranging from 3.2 to 4.6 μM. Treatment of HCT-116 cells with each of the compounds resulted in a depletion of Hsp90 client proteins, CRAF, ERBB2 and CDK4, confirming that antiproliferative activity was linked to the inhibition of Hsp90 activity. Furthermore, such treatment caused upregulation of Hsp27 and Hsp72 expression, suggesting an induction of the heat shock response. Moreover, an increase in PARP cleavage evoked by the tested compounds indicated the induction of apoptosis [8].

### Miscellaneous 1,2,3-thiadiazole derivatives

Aside from the derivatives presented above, there are also some other compounds containing 1,2,3-thiadiazole moiety which display an anticancer activity, but their molecular targets remain unknown. Among a series of d-ring fused 1,2,3-thiadiazole dehydroepiandrosterone (DHEA) derivatives, the most potent compounds 22, 23 and 25 presented antitumor activity against human breast cancer T47D cells with IC_50_ values ranging between 0.042 and 0.058 μM. These values were comparable to that of reference drug adriamycin (IC_50_ = 0.04 μM). Of note, compound 25 possessed a considerable selectivity towards T47D cells. Its activity against the other breast cancer cell lines, MDA-MB-231 and MCF-7, as well as human prostate cancer (DU-145 and LNCaP), colon carcinoma (HCT-116 and HT-29), promyelocytic leukemia (HL-60) and immortalized T lymphocyte (Jurkat) cell lines ranged between 2.49 and 46.0 μM. Moreover, IC_50_ of compound 25 in normal human fibroblast was > 50 μM, while adriamycin presented IC_50_ of 0.068 μM in these cells. Further analysis revealed that compound 25 significantly inhibited the ability of T47D cells to form colonies and migrate as well as induced apoptosis. It also increased the phosphorylation level of ephrin (Eph) receptors, EphA2 and EphB3, proteins involved in the pathogenesis of breast cancer [9]. Clearly further studies are required to elucidate whether the deregulation of EphA2 and EphB3 contributes to the anticancer activity of compound 25 or not. Of note, compound 25 significantly inhibited tumor growth and metastatic ability in T47D xenografts [10].

Another example of derivatives bearing the 1,2,3-thiadiazole ring are pyrazole oxime compounds designed and synthesized by Dai et al. [11]. The activity of these compounds towards human pancreatic cancer Panc-1 cells, hepatocarcinoma Huh-7 cells, colon cancer HCT-116 cells, and gastric cancer SGC-7901 cells was evaluated. The most potent among 23 tested compounds appeared to be compounds 8e and 8l bearing methyl substituent at position 4 of the thiadiazole ring. These compounds differed in terms of substituents in the phenyl ring as compound 8e was 4-bromo substituted, while compound 8l was 2,3-difluoro substituted. In Panc-1 cells each of the compounds displayed anticancer effect (IC_50_ of 12.79 and 12.22 μM, respectively) similar to that of sorafenib (IC_50_ of 11.50 μM). In Huh-7 cells these compounds presented activity (IC_50_ of 11.84 and 10.11 μM, respectively) comparable to that of cisplatin (IC_50_ of 12.70 μM). In HCT-116 cells the anticancer activity of compound 3e and 3l was much stronger (IC_50_ of 7.19 and 6.56μM, respectively) compared to that of 5-fluorouracil (IC_50_ of 29.50 μM). Similarly, in SGC-7901 cells both compounds displayed much stronger activity (IC_50_ of 15.50 and 25.65 μM, respectively) than 5-fluorouracil (IC_50_ of 56.12 μM) [11].

## Derivatives of 1,2,4-thiadiazole

Resveratrol is a naturally occurring *trans* stilbene with anticancer and cancer chemopreventive potential. However, due to rapid and extensive metabolism, its bioavailability is poor. A growing body of evidence indicates that various chemical modifications may significantly improve resveratrol’s bioavailability and potency against different types of cancer [12]. Mayhoub and co-workers replaced the *trans* stilbene ethylenic bridge of the resveratrol scaffold with a 1,2,4-thiadiazole heterocycle and next modified the substituents on the two aromatic rings and evaluated cytotoxicity of these compounds against human breast cancer MCF-7 cell line. These compounds displayed the IC_50_ values ranging from 4.7 to 39.8 μM and the most potent turned out to be 3-hydroxyderivative termed 3jj. Although modifications introduced to the compounds’ structure altered their potency and specificity against three enzymes inhibited by resveratrol, i.e. aromatase, NF-κB and quinone reductase 1, it did not translate to the anticancer activity towards MCF-7 cells [13].

Derivatives based on 3-substituted benzo[4,5]imidazo[1,2-*d*] [1,2,4]thiadiazole linked with a polymethylene spacer to α-bromoacryloyl amido benzoheterocycles are another example of compounds carrying 1,2,4-thiadiazole moiety with an anticancer activity. These compounds showed a considerable activity in human myeloid leukemia cell lines HL-60 and U937 (IC_50_ values from 0.24 to 1.72 μM), as well as in melanoma SK-MEL-1 cell line (IC_50_ from 2.09 to 8.95 μM). The most active were compounds 1 and 2 containing *N*-unsubstituted indole in the benzoheterocycle. Comparing these two derivatives, the anticancer activity decreased on increasing the length of the chain from three (compound 1) to six (compound 2) methylene units. Among the derivatives characterized by the same alkyl chain (6 methylene units), the greatest potency was exhibited by compound 2 bearing α-bromoacrylamidoindole moiety (IC_50_ values from 0.40 to 3.02 μM). A replacement of indole (compound 2) with *N*-methyl indole (compound 3) decreased an anticancer activity. Further decrease was observed when indole was replaced with benzofuran (compound 4) and benzothiophene (compound 5). Treatment with each of compounds induced apoptosis in HL-60 and U937 cells, although most effective anticancer compound 1 appeared to be a less potent apoptosis inducer compared to the rest of derivatives, suggesting that this compound may trigger also some other mechanisms underlying cell death [14].

## Derivatives of 1,2,5-thiadiazole

Anthra[2,1-*c*][1,2,5]thiadiazole-6,11-dione, further termed as NSC745885, turned out to be the most potent among a series of anthra[1,2-*d*]imidazole-6,11-dione tetracyclic analogues with different side chain. Out of 60 cancer cell lines of different origin, the most remarkable sensitivity to NSC745885 displayed leukemia, melanoma, ovarian, breast, prostate cancer as well as glioma cell lines (GI_50_ values between 0.16 and 7.71 μM). The relatively lower, yet significant sensitivity to NSC745885 presented colon and non-small cell lung cancer cell lines (GI_50_ values between 1.28 and 17.40 μM) [15].

**Fig. 3:**
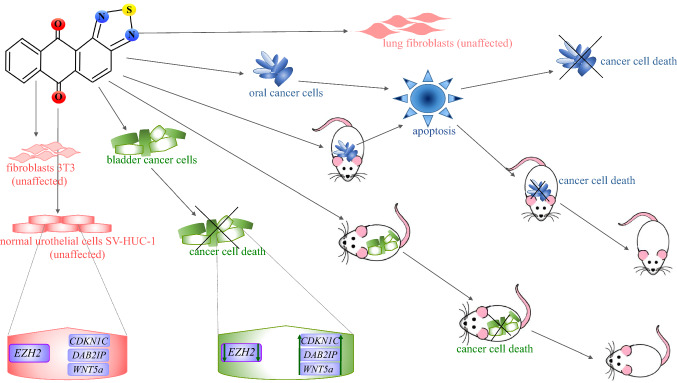
Anticancer activity of NSC745885 (anthra[2,1-c][1,2,5]thiadiazole-6,11-dione). NSC745885 induces apoptosis in oral cancer cells in vitro and in vivo, but does not affect the viability of lung fibroblasts. In bladder cancer cells, NSC745885 downregulates expression of the enhancer of zeste homolog 2 (EZH2), which results in up-regulation of EZH2-silenced tumor suppressor genes, *CDKN1C*, *DAB2IP*, and *WNT5a*. Those alterations contribute to cancer cell death in vitro and in vivo and are not observed in normal fibroblasts 3T3 or urothelial cells SV-HUC-1, which are resistant to NSC745885 treatment

Further analysis revealed that NSC745885 was also effective in oral cancer cell lines, but anticancer concentrations of this compound, up to 4 μM, did not significantly affect the viability of normal lung fibroblasts. Treatment with NSC745885 reduced tumor growth iv vivo, but did not decrease the bodyweight of the animals. This compound displayed anti-tumor efficiency similar to that of doxorubicin, an anthraquinone derivative, but higher safety. Induction of apoptosis was observed upon treatment with NSC745885 both in vitro and in vivo (Fig. 3) [16].

In another study from the same group, 2.5 μM NSC745885 reduced the viability of bladder cancer T24 and MBT2 cell lines and induced G2/M cell cycle arrest, but did not harm immortalized normal urothelial cells SV-HUC-1 or normal fibroblasts 3T3. Of note, the same inhibitory effect on viability was observed when the cancer cells were treated either with 2.5 μM doxorubicin or 40 μM emodin, a natural anthraquinone, but both of these compounds presented significant toxicity towards SV-HUC-1 and 3T3 cells. Moreover, treatment with 2.5 μM NSC745885 suppressed the viability of multi-drug resistant bladder cancer MGH-U1R cells. This compound also displayed a remarkable antitumor activity in vivo. NSC745885 treatment downregulated expression of the enhancer of zeste homolog 2 (EZH2) in cancer cells, but not SV-HUC-1 cells. EZH2 is a member of the polycomb repressive complexes 2 (PRC2) that catalyzes the methylation of histone H3 lysine 27 (H3K27), which in turn mediates chromatin compaction. Overexpression of *EZH2* is observed in numerous cancer of different origin and its inactivation was therapeutically effective in several cancer models [17]. Of note, the other components of PRC2 and the global H3K27 methylation, modified by EZH2, remained unaffected by NSC745885 treatment. The up-regulation of EZH2-silenced tumor suppressor genes, *CDKN1C*, *DAB2IP*, and *WNT5a*, was found in NSC745885-treated cancer cells, but not SV-HUC-1 cells (Fig. 3). More detailed molecular analysis revealed that suppression of the cancer cells’ viability by NSC745885 treatment was indeed causatively linked to downregulation of EZH2 [18].

The same group further investigated anticancer activity of NSC745885 and its 4-chloro derivative, NSC757963. While melanoma and ovarian cancer cell lines were particularly sensitive to NSC745885 (GI_50_ values ranging from 0.55 to 7.71 μM), leukemia cell lines were notably susceptible to NSC757963 (GI_50_ values from 0.33 to 3.15 μM). Colon and non-small cell lung cancer cell lines presented the lowest sensitivity to NSC745885 (GI_50_ values from 1.28 to 19.00 μM) and NSC757963 (GI_50_ values from 1.43 to > 100 μM). Either of compounds inhibited translocation of NF-κB to the nucleus, which in turn suppressed constitutive activation of this transcription factor. Docking studies revealed favorable binding of both compounds to the ATP site of the N-terminal kinase domain of the IKKβ subunit, a very potent activator of NF-κB [19].

In a subsequent analysis, the same group examined an anticancer activity of a series of sulfur-substituted anthra[1,2-*c*][1,2,5]thiadiazole-6,11-dione derivatives. Among these compounds, 4-(isopropylthio)anthra[1,2-*c*][1,2,5]thiadiazole-6,11-dione, termed NSC763968, appeared to be most active. Leukemia and prostate cancer cell lines were particularly sensitive to this compound (GI_50_ values ranging from 0.18 to 1.45 μM). Slightly lower toxicity of NSC763968 was observed in ovarian cancer, breast cancer, melanoma, renal cancer, glioma and non-small cell lung cancer cell lines (GI_50_ values from 0.20 to 5.68 μM). Colon cancer cell lines were the least sensitive to NSC763968 (GI_50_ values ranging from 0.29 to 13.30 μM). Of note, 10 μM concentration of this compound was relatively not toxic towards human non-cancerous cell lines, as it reduced the viability of urothelial SV-HUC-1 cells to 80%, prostatic stromal myofibroblasts WMPY-1 to 60%, and prostate epithelial cells RWPE-1 to 90%. At the same time, 10 μM doxorubicin decreased the viability of SV-HUC-1, WMOY-1 and RWPE-1 cells to 50%, 20%, and 40%, respectively. In prostate cancer DU-145 cells, NSC763968 induced apoptosis and inhibited phosphorylation of ERK and p38 kinases [20]. As both ERK and p38 pathways are involved in the pathogenesis of cancers of different origin [21, 22], it is tempting to speculate that NSC763968 treatment inhibits those pathways, which in turn contributes to the anticancer activity of this compound.

In very recent research from the same group, a nitrogen-substituted anthra[1,2-*c*] [1,2,5]thiadiazole-6,11-dione derivative, RV-59, turned out to be particularly toxic to human colon cancer HCT116 cells with high cytoplasmic level of a transcription factor Nrf2 (cNrf2). A significant contribution of a cytoplasmic, but not nuclear, localization of Nrf2 to aggressive phenotype of colorectal cancer cells has previously been documented by the same group [23]. The IC_50_ value of RV-59 was 3.55 μM for HCT116 cells with high cNrf2, and 16.81 μM for Nrf2-knockdown HCT116 cells. The IC_50_ value of 5-FU, a drug used to treat colon cancer [24], was 17.74 μM for the cells with high cNrf2, and 5.35 μM for the cells not expressing Nrf2. These results indicate that RV-59 predominantly kills and overcomes cNrf2-mediated resistance to 5-FU. Further analysis clearly showed that RV-59 remarkably suppressed xenograft tumor growth induced by the cells with cNrf2-mediated 5-FU resistance. Of note, RV-59 treatment did not affect the body weights of the animals, suggesting that this drug may not be toxic to normal cells [25].

## Derivatives 1,3,4-thiadiazole

### Inhibitors of inosine monophosphate dehydrogenase (IMPDH)

One of the first studies documenting anticancer activity of 1,3,4-thiadiazole derivatives was published by Shapiro et al. in 1957 [26]. In this paper, 2-ethylamino-1,3,4-thiadiazole (EATDA), an analog of niacin, inhibited the growth of mammary adenocarcinomas induced in mice. The tumor-inhibitory effect of this compound was prevented by the prior injection of nicotinamide, supporting the evidence that it is a niacin antagonist. Moreover, the addition of EATDA to the combination of 8-azaguanine, deoxypyridoxine and testosterone improved anticancer activity of this three-drug combination [26].

**Fig. 4:**
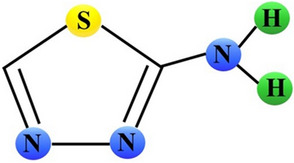
Schematic chemical structure of 2-amino-1,3,4-thiadiazole (ATDA; NSC 4728). Sulphur, nitrogen, and hydrogen atoms are marked as yellow, blue or green circles, respectively

EATDA and 2-amino-1,3,4-thiadiazole (ATDA) displayed anti-cancer properties in mice surviving systemic leukemia L1210. These compounds not only inhibited tumor growth but also prolonged survival time of the animals. The anti-leukemic activity and host toxicity of either compound were blocked by the administration of nicotinamide [27]. Later study revealed that treatment of L1210-bearing mice with ATDA, further referred to as NSC 4728 (Fig. 4), diminished adenine and guanine ribonucleotide pools and increased uridine triphosphate (UTP) and inosine monophosphate (IMP) pools in the tumor cells. This effect was prevented by simultaneous administration of nicotinamide. Taking into account that nicotinamide prevents anti-leukemic activity of ATDA, it might suggest that the inhibition of guanosine monophosphate synthesis was related to the anti-leukemic action of this compound [28]. In further mechanistic studies, NSC 4728 and its derivatives appeared to be potent inhibitors of IMP dehydrogenase (EC 1.2.1.14), an enzyme involved in the conversion of IMP to xanthosine monophosphate (XMP), a substrate for the production of guanosine monophosphate (GMP) (Fig. 2) [29].

In phase I clinical trials, NSC 4728 was administered daily, twice a week or weekly in a range of doses from 2 to 200 mg/m^2^ to 42 patients suffering from different cancers. One patient with squamous cell carcinoma of the lung and one with squamous cell carcinoma of the cervical esophagus exhibited a partial response to a total dose of 550 or 575 mg/m^2^, respectively. In either patient, a progression of disease was observed after 2 months despite continued treatment. In phase II, 125 mg/m^2^ of NSC 4728 was given once a week to 29 patients with non-small cell lung carcinoma. A partial response was observed in one patient after receiving a total of 1200 mg/m^2^ and another one after two doses of 125 mg/m^2^ of NSC 4728. In either patient, the progression was observed despite continued treatment. Stomatitis was the most common adverse effect, but dermatitis, nausea vomiting, lethargy, and hyperuricemia were observed as well [30].

Out of 46 patients with metastatic renal cell carcinoma one patient, treated with NSC 4728 in a daily dose of 125 mg/m^2^, experienced partial remission. Leukopenia, thrombocytopenia and anemia were the most common side effects in patients enrolled in this phase II clinical trials [31].

Asbury and co-workers conducted several phase II clinical trials of NSC 4728 in a dose of 125 mg/m^2^ weekly in patients with different tumor types. Partial response to treatment was observed in 12% of patients with advanced colon cancer. Gastrointestinal toxicity was severe in l6% of patients [32]. A partial response was also observed in 5% of patients with squamous carcinoma of the cervix and 28% of patients had stable disease. The patients exhibited mild renal toxicity and a single life-threatening toxic episode was observed [33]. Complete response was observed in 8% of patients with non-squamous cervical carcinoma, and 35% had stable disease. Anemia and emesis were the main side effects [34]. Partial response was exhibited by 5% of patients with mixed mesodermal tumors of the uterine corpus. Severe nausea and anemia were often [35]. No response was observed in patients with either advanced or recurrent leiomyosarcoma [36] or advanced epidermoid carcinoma of the esophagus [37] treated in the same way. Clinical trials with NSC 4728 are summarized in Table 1.

### Inhibitors of carbonic anhydrase (CA)

Some of the 1,3,4-thiadiazole derivatives turned out to inhibit the activity of human carbonic anhydrase (CA; EC 4. 2. 1. 1). This zinc metalloprotein catalyzes CO_2_/HCO_3_^−^ interconversion and is thereby involved in several physiological and pathological processes. So far, 14 humans CA isoforms have been identified and each of them serves as biomarker for various diseases, including cancers of several origin [38].

**Fig. 5:**
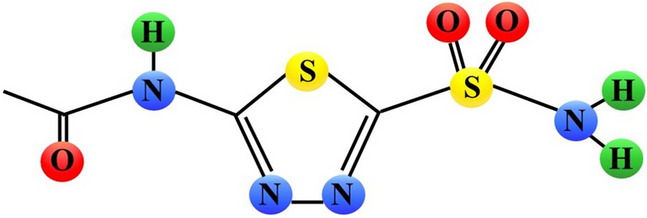
Schematic chemical structure of acetazolamide (5-acetamido-1,3,4-thiadiazole-2-sulfonamide, AZA). Sulphur, nitrogen, hydrogen, and oxygen atoms are marked as yellow, blue, green or red circles, respectively

A CA inhibitor acetazolamide (5-acetamido-1,3,4-thiadiazole-2-sulfonamide, AZA) (Fig. 2, Fig. 5) displaying diuretic effects is already used in clinics. There are two currently ongoing phase I clinical trials with AZA (Diamox) in cancer patients. In one of them AZA in combination with platinum and etoposide-based radiochemotherapy is evaluated in patients with small-cell lung cancer (NCT03467360). In the other one, AZA in combination with temozolomide is assessed in patients with malignant glioma (NCT03011671) (Table 1).

Supuran and Scozzafava documented inhibitory effect of the other 1,3,4-thiadiazole-2-sulfonamide derivatives on CA isoforms [39]. Nanomolar concentrations of compounds 10–13 remarkably inhibited the activity of CA II and CA IV but were less potent towards CA I. Nanomolar concentrations of the urea/thiobiguanide derivatives 14–16 inhibited CA I and CA II and to the lesser extend CA IV. Most of these compounds were more potent CA inhibitors than AZA. Compounds 14–16 were much more cytotoxic (GI_50_ values from 12 to 70 μM) than compounds 10–13 which in most cancer cell lines displayed GI_50_ values > 100 μM. The exception was the OVCAR-4 ovarian cancer cell line, susceptible to compounds 10 and 11 (GI_50_ values of 0.5 and 0.1 μM, respectively). The exact mechanism underlying anticancer activity of these derivatives was not elucidated, but the authors postulated that the acidification of the intracellular environment resulted from CA inhibition might be of crucial importance [39].

In the later study from the same group, compound 14, a biphenyl-disulfonamide derivative bearing 5-amino-1,3,4-thiadiazole-2-sulfonamide, inhibited CA II and CA IX, and to the lesser extend also CA XII and CA I. This compound displayed cytotoxicity towards human colon cancer HCT116 cell line with GI_50_ value of 3.789 μg/mL but was less potent against non-small cell lung cancer H460 and breast cancer MCF7 cells [40].

### Inhibitors of glutaminase (GA)

Glutamine (Gln) plays the versatile and crucial role in tumors regardless of the driving oncogene or tissue of origin. Increased metabolism of Gln, of which the first step is catabolized by glutaminase (GA; EC 3.5.1.2), is a hallmark of cancer. Therefore, GA is a potential therapeutic target for different cancer types [41].

Treatment with 2 μM 10 μM of bis-2-(5-phenylacetamido-1,3,4-thiadiazol-2-yl)ethyl sulfide (BPTES), a specific inhibitor of kidney type GA (GLS), diminished the proliferation of human Burkitt lymphoma P493 cells [42]. Higher concentrations (10–40 μM) of this compound decreased the viability of glioblastoma [43], non-small cell lung cancer [44] and acute myeloid leukemia [45] cells. Moreover, BPTES injected daily in a dose of 12.5 mg/kg body weight inhibited lymphoma and hepatocellular carcinoma xenograft growth [42, 46]. Poor drug-like molecular properties of BPTES, mainly extremely poor aqueous solubility (< 1 μg/mL) ruled out the feasibility of further development of this compound as a therapeutic agent. Of a number of BPTES-derived GLS inhibitors displaying much better drug-like properties compared to BPTES recently synthesized [47], only those already tested in cancer models will be presented below.

A truncated analog of BPTES, *N*-(5-{2-[2-(5-Amino-[1,3,4]thiadiazol-2-yl)-ethylsulfanyl]-ethyl}-[1,3,4]thiadiazol-2-yl)-2-phenyl-acetamide, referred to as compound 6, exhibited potency similar to that of BPTES, but much better aqueous solubility of 13 μg/mL. Treatment with 20 μM concentration of this compound significantly attenuated the growth of lymphoma B P493 cells in vitro as well as in a mouse xenograft model while injected daily in doses of 12.5 mg/kg [48].

**Fig. 6:**
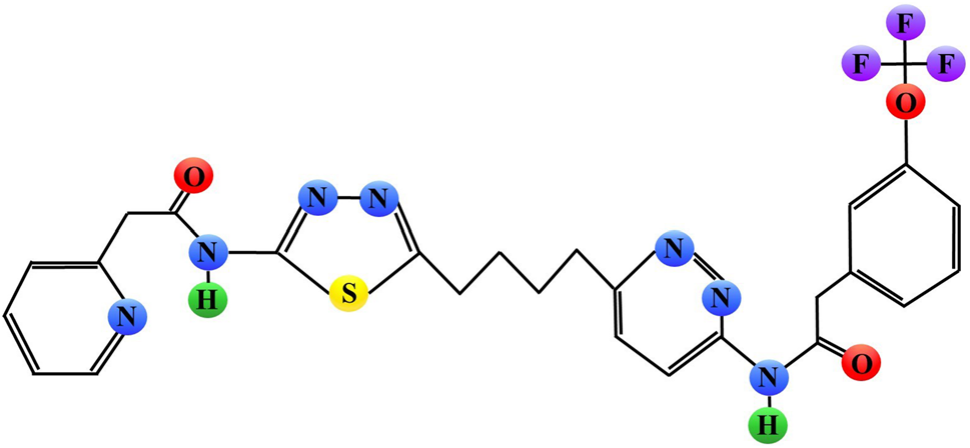
Schematic chemical structure of CB-839 (*N*-[5-[4-[6-[[2-[3-(trifluoromethoxy)phenyl]acetyl]amino]-3-pyridazinyl]butyl]-1,3,4-thiadiazol-2-yl]-2-pyridineacetamide). Sulphur, nitrogen, hydrogen, oxygen or fluorine atoms are marked as yellow, blue, green, red or purple circles, respectively

Gross and co-workers discovered *N*-[5-[4-[6-[[2-[3-(trifluoromethoxy)phenyl]acetyl]amino]-3-pyridazinyl]butyl]-1,3,4-thiadiazol-2-yl]-2-pyridineacetamide, referred to as CB-839 (Fig. 6). Its IC_50_ value for GA inhibition (Fig. 2) was < 50 nmol/L, 13-fold lower than that of BPTES. CB-839 treatment inhibited the proliferation of the triple-negative breast cancer (TNBC) cell lines, HCC1806 and MDA-MB-231, (IC_50_ of 49.0 and 26.0 nmol/L, respectively), but had no effect on the viability of the breast cancer ER^+^/HER2^−^ cell line T47D. The rates of Gln consumption were reduced for HCC1806 and MDA-MB-231 cells treated with CB-839, indicating that the antitumor activity of this compound was indeed linked to the diminished Gln metabolism. The in vivo efficacy of CB-839 was further examined in two breast cancer xenograft models, a primary patient-derived TNBC xenograft and an HER2^+^ basal-like cell line JIMT-1-based xenograft. In either model, oral dosing of 200 mg/kg of CB-839 twice daily remarkably inhibited xenografts’ growth and was well tolerated, with no overt signs of toxicity [49].

In *EGFR* mutant non-small cell lung cancer HCC827 cells, treatment with 300 nM CB-839 resulted in approximately 50% reduction of the viability. Administration of this compound twice daily in a dose of 200 mg/kg inhibited xenograft growth. Furthermore, the combination of an EGFR inhibitor, erlotinib, and CB-839 cooperated to inhibit the growth of HCC827 cells in vitro and in vivo [50]. For the other lung cancer cell lines, A427, A549 and H460, the CB-839 ED_50_ values for inhibition of colony formation were 9.1, 27.0 and 217 nM, respectively. Treatment with 1 μM CB-839 increased response of H460 cells to radiation. The short-term CB-839 dosing at 200 mg/kg did not affect xenograft tumor growth, but the combination of CB-839 administration with the radiation dose of 12 Gy reduced tumor growth by 15–30% [51].

Treatment with 500 nM CB-839 inhibited the viability of *NF1* mutant/null malignant peripheral nerve sheath tumor cell lines (MPNST), ST8814 and S462. Administration of 200 mg/kg of CB-839 significantly suppressed the volume of MPNST xenografts [52]. In chondrosarcoma cell lines, CB-839 activity correlated with the status of isocitrate dehydrogenase 1/2 (IDH1/2), as the cells carrying *IDH1/2* mutation were more susceptible to this compound than the wild type cells [53]. In a very recent study, CB-839 administered twice daily at dose 200 mg/kg significantly reduced undifferentiated pleomorphic sarcoma (UPS) tumor growth and weight but did not alter animal weights [54].

Taken together, data presented above clearly indicate that CB-839 exhibits high therapeutic potential. Indeed, this drug was evaluated either as a single agent in patients with leukemia (NCT02071927), hematologic malignancies (NCT02071888), and solid tumors (NCT02071862), as well as in combination with paclitaxel in patients with triple-negative breast cancer (NCT03057600) or in combination with panitumumab and irinotecan in patients with colorectal cancer (NCT03263429). However, detailed results of any of those trials are not yet available. Numerous clinical trials evaluating anticancer activity of CB-839 are still ongoing (Table 1).

Recently, 2-phenyl-*N*-(5-(4-((5-(2-phenylacetamido)-1,3,4-thiadiazol-2-yl)amino)piperidin-1-yl)-1,3,4-thiadiazol-2-yl)acetamide (UPGL00004), another analog of BPTES, turned out to be a promising anti-cancer agent. In this compound, previously termed 7c, the flexible region of BPTES or CB-839 has been replaced by relatively rigid heterocyclic core [55]. UPGL00004 inhibited the enzymatic activity of GLS more potently than BPTES and displayed binding affinity for this protein similar to that of CB-839 (Fig. 2). Crystallographic studies revealed that UPGL00004 occupied the same binding site as CB-839 or BPTES and that all three compounds inhibited the enzymatic activity of GLS via a similar allosteric mechanism. The anticancer activity of UPGL00004 was examined in a triple-negative breast cancer patient-derived tumor graft model. Neither UPGL00004 in a dose 1 mg/kg body weight nor approved for the treatment of metastatic breast cancer bevacizumab in a dose 2.5 mg/kg body weight reduced tumor growth. However, a combination of these compounds completely prevented an increase in tumor size during the course of treatment [56].

### Inhibitors of histone deacetylase (HDAC)

Histone deacetylase (HDAC, EC 3.5.1.98) removes acetyl groups from DNA-binding histone proteins, which in turn decreases chromatin accessibility for transcription factors and blocks the transcription. The human HDAC family consists of 18 proteins divided into 4 classes. These proteins modulate, among others, the transcription of genes encoding proteins involved in carcinogenesis [57].

Rajak and co-workers designed and synthesized a series of 2-[5-(4-substitutedphenyl)-[1, 3, 4]-thiadiazol-2-ylamino]-pyrimidine-5-carboxylic acid hydroxyamides, which exhibited the HDAC1 inhibitory activity with IC_50_ values between 0.008 and 0.018 μM (Fig. 2). Treatment with these compounds decreased the viability of human colorectal carcinoma HCT-116 cells (IC_50_ values ranging from 0.08 to 0.31 μM). Moreover, each of the compounds administered at seven doses of 0.2 mmol/kg significantly inhibited weight and growth of tumors formed by Ehrlich ascites carcinoma (EAC) cells inoculated into mice. The antitumor activity of compounds changed on varying *para*-substituted group on aryl moiety attached to 1,3,4-thiadiazole as follows: hydroxy > methoxy > methyl > amino > dimethylamino > nitro > chloro > fluoro > no substitution [58].

Guan and co-workers designed and synthesized a series of amino-1,3,4-thiadiazole-based hydroxamic acid derivatives with different linkers and substitution in thiadiazole ring. Both the length of the linker chain and the substitution in 1,3,4-thiadiazole turned out to be important for the HDAC inhibitory activity. Compounds with the linker comprised of five or six methylene units inhibited HDAC in the nanomolar range, while the rest derivatives showed only micromolar activity. Compounds with the phenyl or benzyl substitution in 1,3,4-thiadiazole were more potent than the those substituted with phenethyl or (E)-styryl. The linker between zinc-binding group and 1,3,4-thiadiazole ring was more important for the anti-HDAC activity than the substitutions in 1,3,4-thiadiazole moiety. Compound 6i presented an increased HDAC inhibitory activity (IC_50_ 0.089 μM) compared to that of suberoylanilide hydroxamic acid (SAHA), an HDAC inhibitor (IC_50_ 0.15 μM). Docking studies revealed that this compound had a similar binding mode to SAHA in the active site of HDAC1. Treatment with each of four most potent HDAC inhibitors (IC_50_ 0.089–0.26 μM) decreased the viability of the human breast cancer MDA-MB-231 cells (IC_50_ 2.98–6.14 μM) as well as chronic myelogenous leukemia K562 cells (IC_50_ 6.75–12.9 μM). The IC_50_ values displayed in MDA-MB-231 and K562 cells by SAHA were 1.32 and 1.69 μM, respectively [59].

In the next study, the same group attempted to increase the anticancer activity of 1,3,4-thiadiazole bearing hydroxamates. Substitution of the phenyl ring did not increase the HDAC inhibitory activity. Three important findings appeared from this part of the experiments: i. phenyl substitution with a bulky group resulted in the loss of the HDAC inhibitory activity; ii. compounds with an electron-withdrawing group exhibited poorer HDAC inhibition than those with an electron-donating group; iii. following tendency in enzymatic potency was found: para-< meta-< ortho-substitution. Replacement of the phenyl ring with a naphthalenyl group led to a loss in HDAC inhibition, while analogues containing pyridine or thiophene displayed higher or similar activity (IC_50_ 286–411 nM) compared to SAHA (IC_50_ 416 nM). Compound 35 bearing thiophen-2-yl and 6 carbon units in the linker appeared to be the more active against three cancer cell lines, MDA-MB-231, K562 and human prostate cancer PC3 (IC_50_ 1.21, 1.56 and 3.6 μM, respectively) compared to SAHA (IC_50_ 2.29, 1.61 and 5.79 μM, respectively) [60].

A series of 5-substitutedphenyl-1,3,4-thiadiazole-based hydroxamic acids was also evaluated by Nam and co-workers [61]. In this study, compound 5a, of which the HDAC inhibitory activity (Fig. 2), but not cytotoxicity has been previously examined [59], showed strong cytotoxicity against human colon cancer SW620, breast cancer MCF7, prostate cancer PC3, pancreas cancer AsPC1 and lung cancer NCI-H460 cells (IC_50_ 0.70, 1.80, 0.88, 2.71, 1.07 μM, respectively). Analogs bearing one halogen atom on the phenyl ring, either at position 2, 3 or 4, presented the cytotoxicity comparable or slightly higher to that of compound 5a. Substitution of a chlorine atom at position 2 was the most favorable for cytotoxicity (IC_50_ between 0.11 and 1.23 μM). In the same assay, SAHA displayed IC_50_ values between 2.77 and 6.42 μM. Of note, the introduction of an additional chlorine at position 6 or nitro substituent at position 2 or 4 remarkably decreased cytotoxicity. Western blot analysis revealed that treatment with compounds showing cytotoxicity comparable or higher to that of SAHA increased level of histone acetylation, suggesting that the anticancer activity of those compounds might be linked to their HDAC inhibitory activity. Results of docking studies indicated that two compounds 5b (bearing 2-chlorophenyl) and 5c (bearing 3-chlorophenyl) had higher binding affinities to HDAC8 compared to SAHA [61].

### Inhibitors of kinesin spindle protein

The mitotic kinesins are the proteins responsible for force generators in the process of cell division. The most extensively studied of these proteins is Eg5 (also known as KIF11, kinesin-5 or KSP). Due to its role in cell division, Eg5 is a potential cancer-selective therapeutic target. Indeed, overexpression of this protein is observed in tumors of different origin [62].

Based on the results of a phenotype-based forward chemical genetics screen, Nakai and co-workers selected K858 (*N*-(4-Acetyl-4,5-dihydro-5-methyl-5-phenyl-1,3,4-thiadiazol-2-yl)acetamide) as an antimitotic agent. This compound induced mitotic arrest, caspase-dependent apoptosis, and cell growth inhibition in human colon cancer HCT-116 cells, but had no effect on microtubule polymerization. K858 blocked centrosome separation and induced the formation of a monopolar spindle during mitosis as well as inhibited the ATPase activity of Eg5 with an IC_50_ of 1.3 μM. Of note, this compound appeared to be 150-fold more selective for Eg5 than other members of the kinesin superfamily. It should be also emphasized that K858 induced mitotic cell death in HCT-116 cells, but not in non-cancerous retinal pigment epithelial ARPE-19 cells. Moreover, treatment of mice with 150 mg/kg K858 suppressed tumor growth in a human A2780 ovarian cancer model but no overt evidence of toxicity was found [63].

K858 and its derivative bearing an ethyl moiety at C5 position of the thiadiazole ring, compound 33, displayed significant antitumor activity against human prostate cancer PC3 and melanoma SK-MEL-5 and SK-MEL-28 cells. Both K858 and compound 33 presented the ability to inhibit Eg5 enzymatic activity (Fig. 2) [64]. K858 diminished the viability and induced apoptosis in human breast cancer MCF7, MDA-MB231, BT474 and SKBR3 cells [65] as well as in human glioblastoma U87 and U251 cells [66].

**Fig. 7:**
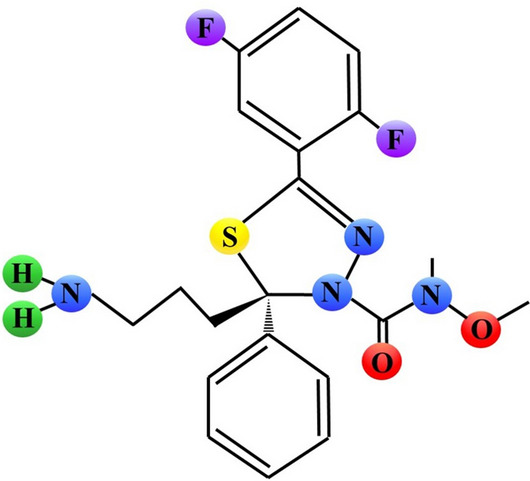
Schematic chemical structure of filanesib ((2*S*)-2-(3-Aminopropyl)-5-(2,5-difluorophenyl)-*N*-methoxy-*N*-methyl-2-phenyl-1,3,4-thiadiazole-3(2*H*)-carboxamide trifluoroacetate, ARRY-520). Sulphur, nitrogen, hydrogen, oxygen or fluorine atoms are marked as yellow, blue, green, red or purple circles, respectively

The enzymatic activity of Eg5 is also blocked by the other 1,3,4-thiadiazole derivative, (2*S*)-2-(3-Aminopropyl)-5-(2,5-difluorophenyl)-*N*-methoxy-*N*-methyl-2-phenyl-1,3,4-thiadiazole-3(2*H*)-carboxamide trifluoroacetate, termed Filanesib or ARRY-520 (Fig. 2, Fig. 7). Treatment of leukemic U937, Jurkat, and HL-60, Molm13 and OCI-AML3 cells with nanomolar concentrations of this compound induced G2/M cell cycle block and cell death. This compound (at dose 27 mg/kg) significantly inhibited tumor growth of HL-60 xenografts in mice. Moreover, it diminished the colony formation capacity of blasts from patients with acute myeloid leukemia but not normal blood cells [67]. A similar regimen inhibited volume of xenografts formed by colon cancer HT-29, breast cancer UISO-BCA-1, prostate cancer PC3 and myeloma RPMI8226, JJN3, U266, and NCI H929 cells [68, 69].

Filanesib has already been evaluated in clinical trials in patients with different cancers (summarized in Table 1). Filanesib demonstrated an acceptable safety profile at dose levels up to 4.5 mg/m^2^ in patients with advanced myeloid leukemia (AML). Partial response was observed in 3% and stable disease in 28% of patients. Drug-related serious adverse events, mainly mucositis and neutropenic fever, were observed in 28% of patients and led to study discontinuation in 8% [70]. No partial or complete response was noted in patients with advanced solid tumors treated with filanesib at dose levels up to 2.50 mg/m^2^ [71].

In a cohort of 55 patients with multiple myeloma (MM), a combination of filanesib plus bortezomib and dexamethasone demonstrated a favorable safety profile. The overall response rate (ORR) was 20%, the clinical benefit rate (CBR) was 33%, and the disease control rate was 65% [72]. Later, filanesib 1.50 mg/m^2^/day alone or in combination with dexamethasone was evaluated in phase 2 trials in 25 patients with MM. Filanesib has single-agent an ORR of 16% and a clinically meaningful CBR of 23%. The response rates in filanesib/dexamethasone population were also clinically relevant (ORR 15%; CBR 20%) [73]. The results of the most recent clinical trials with filanesib in patients with MM remain unknown.

**Fig. 8:**
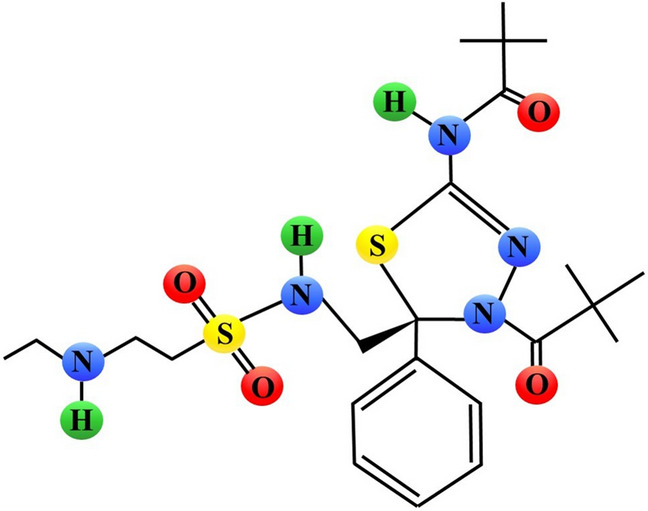
Schematic chemical structure of litronesib (*N*-[(5R)-4-(2,2-dimethylpropanoyl)-5-[[2-(ethylamino)ethylsulfonylamino]methyl]-5-phenyl-1,3,4-thiadiazol-2-yl]-2,2-dimethylpropanamide, LY2523355). Sulphur, nitrogen, hydrogen and oxygen atoms are marked as yellow, blue, green or red circles, respectively

More recently, another Eg5 inhibitor, *N*-[(5R)-4-(2,2-dimethylpropanoyl)-5-[[2-(ethylamino)ethylsulfonylamino]methyl]-5-phenyl-1,3,4-thiadiazol-2-yl]-2,2-dimethylpropanamide, referred to as litronesib or LY2523355 (Fig. 2, Fig. 8), has been discovered and characterized. Treatment with this compound resulted in a dose-dependent mitotic arrest of HCT-116 cells and subsequent cell death. Furthermore, LY2523355 showed marked antitumor activity in most of the xenograft tumor models, including patient-derived xenografts [74].

In phase I of trials in patients with solid tumors (NCT01358019), the recommended dose of litronesib was determined to be 5 mg/m^2^/day. No tumor responses were observed in this study [75]. In more recent trials, partial response to litronesib plus pegfilgrastim was observed in 2% of patients with advanced solid tumors and 20% of patients maintained stable disease (NCT01214629; NCT01214642) [76]. Phase II trials evaluating litronesib in patients with different cancer types (NCT01416389; NCT01025284; NCT01059643) have recently been completed and are summarized in Table 1.

### Inhibitors of tubulin polymerization

Kamal and co-workers synthesized a series of compounds with imidazothiadiazole linked with a 3,4,5‐trimethoxyphenyl ring, an indolinone ring, and a phenyl group. These imidazo[2,1‐*b*][1,3,4]thiadiazolindolin‐2‐ones showed considerable cytotoxicity, with IC_50_ values ranging from 1.1 to 8.9 μM against human lung A549, cervical HeLa, breast MCF‐7, and colon HCT-116 cancer cell lines. Among them, compounds 7 ((*E*)‐5‐fluoro‐3‐((6‐*p* ‐tolyl‐2‐(3,4,5‐trimethoxyphenyl)‐imidazo[2,1‐b][1,3,4]thiadiazol‐5‐yl)methylene)indolin‐2-one) and 11 ((*E*)‐3‐((6‐*p* ‐tolyl‐2‐(3,4,5‐trimethoxyphenyl)imidazo[2,1‐b][1,3,4]thiadiazol‐5‐yl)methylene)indolin‐2‐one) appeared to be most potent with IC_50_ values ranging from 1.1 to 1.6 μM and from 2.5 to 2.9 μM, respectively. Compounds 7 and 11 presented anti-tubulin polymerization activity with IC_50_ of 0.15 and 1.23 μM, respectively (Fig. 2). Treatment of A549 cells with either of these compounds decreased the level of a polymerized fraction of tubulin and increased the level of its soluble fraction. Docking studies showed that compounds 7 and 11 bound in the colchicine binding site of polymerized tubulin [77].

The same group synthesized a series of conjugates with a core unit of imidazothiadiazole linked with a cyclopropyl ring, an oxindole moiety and an aryl ring. Among these conjugates, compounds 7 ((*E*)-3-((2-cyclopropyl-6-(4-methoxyphenyl)imidazo[2,1-b] [1,3,4]thiadiazol-5-yl)methylene)indolin-2-one), 14 (((*E*)-3-((6-(4-chlorophenyl)-2-cyclopropylimidazo[2,1-b][1,3,4]thiadiazol-5-yl)methylene)-5-methoxyindolin-2-one) and 15 ((*E*)-5-chloro-3-((6-(4-chlorophenyl)-2-cyclopropylimidazo[2,1-b][1,3,4]thiadiazol-5-yl)methylene)indolin-2-one) exhibited cytotoxicity towards the cell lines used in the previous study with GI_50_ values from 0.13 to 3.8 μΜ. They also displayed the anti-tubulin activity (IC_50_ between 2.8 and 5.6 μM). Treatment with these compounds decreased the level of polymerized fraction of tubulin, induced the cell cycle arrest in the G2/M phase and apoptosis. Similarly to the compounds described in the earlier report, also conjugates 7, 14 and 15 of this study bound in the colchicine-binding site of the tubulin [78].

### Abl kinase inhibitors

The Abelson tyrosine kinase (Abl) regulates cytoskeletal dynamics, organelle trafficking, cell proliferation and survival. Its contribution to the initiation and progression of leukemia is relatively well understood, but recent studies indicate the involvement of this protein in pathogenesis of solid tumors as well [79]. Some of the Abl inhibitors turned out to inhibit also Src kinase, the other protein involved in tumor pathogenesis [80].

Radi and co-workers synthesized a series of substituted benzoylamino-2-[(4-benzyl)thio]-1,3,4-thiadiazole which turned out to inhibit either both Abl and Src kinases, or only one of them. The Abl inhibitory activity of those compounds ranged between 0.044 and 1.26 μM, and their Src inhibitory activity was between 0.064 and 1.137 μM (Fig. 2). In the same assay, imatinib, used as a reference drug, displayed inhibitory activity with IC_50_ of 0.013 and 31 μM towards Abl and Src, respectively. The most potent Abl inhibitor, 5-[(4-fluorobenzoyl)amino]-2-[(4-fluorobenzyl)thio]-1,3,4-thiadiazole, referred to as compound 6a, significantly reduced the clonogenic activity (LD_50_ 2.2 μM) of distinct clones of myeloid progenitors, varying with respect to sensitivity to Gleevec. Compound 6a diminished proliferation of leukemia HL-60 cells and induced their differentiation [81].

More recently, *N*-(5-Nitrothiazol-2-yl)-2-((5-((4-(trifluoromethyl)phenyl)amino)-1,3,4-thiadiazol-2-yl)thio)acetamide, termed as compound 2, turned out to inhibit Abl kinase with an IC_50_ value of 7.4 µM. Docking studies revealed the role of the distal nitro group in the formation of a crucial hydrogen bond with the key amino acid residues of Abl protein. Treatment with this compound inhibited the viability of human leukemia K562, MT-2, Jurkat and cervical carcinoma HeLa cells with IC_50_ values of 33.0, 166.8, 17.9 and 12.4 μM, respectively. In the same assay, imatinib displayed IC_50_ values of 5.0, 9.7, 6.7 and 15.2 μM, respectively. Compound 2 turned out to be 5 times less toxic towards peripheral blood mononuclear cells (PBMC) (IC_50_ 141.3 μM) than imatinib (IC_50_ 28.3 μM). Aside from the inhibitory effect on Abl kinase, compound 2 diminished to the lesser extent activity of BTK, CSK, FYN A, and LCK kinases [82].

### Lipoxygenase inhibitors

Lipoxygenases (LOX, EC 1.13.11) catalyze the oxygenation of the polyunsaturated fatty acids (PUFAs) to form the hydroxyeicosatetraenoic acids (HETEs). Among these enzymes, 15-lipoxygense-1 (15-LOX-1) was found to be involved in the pathogenesis of tumors of different origin, therefore, its targeting may contribute to cancer treatment [83].

Aliabadi and co-workers synthesized a series of compounds bearing 1,3,4-thiadiazole and phthalimide residues and examined the inhibitory effect of these compounds towards 15-LOX-1 (Fig. 2). Compound 4d with *meta* positioning of the methoxy group afforded the highest inhibitory effect (38%) but did not display a remarkable activity against human prostate cancer PC3, colon adenocarcinoma HT29 or neuroblastoma SKNMC cells. The most toxic towards HT29 cells (IC_50_ 10.91 μM) was compound 4f with *ortho* positioning of the fluorine atom. This compound inhibited the activity of 15-LOX-1 in 31%. Compound bearing the fluorine substituent at *meta*-position displayed some cytotoxicity in SKNMC cells (IC_50_ of 50.2 μM) and inhibited the activity of 15-LOX-1 in 26%. Nitro containing derivatives (4a, 4b, 4c) and compound 4 k with *para* positioning of the chlorine substituent did not show any inhibitory activity against 15-LOX-1, although the latter was cytotoxic towards HT29 and SKNMC cells (IC_50_ of 24.06 and 69.7 μM, respectively) [84].

In a recent study, the same group synthesized a series of *N*-(5-(pyridin-2-yl)-1,3,4-thiadiazol-2-yl)benzamide derivatives. Among them, compounds 4j (*o*-methoxy) and 4 k (*m*-methoxy) displayed the best inhibitory activity towards 15-LOX-1 (28 and 26%, respectively). Compound 4j turned out to be more toxic to PC3, HT29 and SKNMC cell lines with IC_50_ values of 4.96, 16.00 and 15.28 μM, respectively [85].

### Compounds interacting with DNA

Numerous drugs, including those displaying anticancer properties, exert their activity by binding to DNA [86]. Ibuprofen and ciprofloxacin, two commercially available drugs, display the ability to bind to DNA, but hybridization of either compound with 1,3,4-thiadiazoles increases this ability. A hybrid molecule of ciprofloxacin and 1,3,4-thiadiazole, ({(3-(5-amino-1,3,4-thiadiazol-2-yl)-1-cyclopropyl-6-fluoro-7-(piperazin-1-yl)quinolin-4(1H)-one)}), termed compound 2, exhibits greater binding constant than ibuprofen linked with 1,3,4-thiadiazole ({(5-(1-(4-isobutylphenyl)ethyl)-1,3,4-thiadiazol-2-amine)}), termed compound 1. Both derivatives display anticancer activity towards human hepatocellular carcinoma Huh-7 cells with IC_50_ values of 64.90 and 25.75 μM for compound 1 and 2, respectively. These results suggest that increased DNA binding correlates with enhanced anticancer properties of compound [87].

The hybrids of 1,3,4-thiadiazole and chalcone containing phenolic moiety are the other example of molecules binding to DNA. These derivatives exerted the strong cytotoxic activity against leukemia HL-60 cells with IC_50_ values in a range from 6.92 to 16.35 μM. Some of the compounds, termed 5a, 5f, 5 h, 5 l, and 5 m, presented cytotoxicity towards cervical cancer HeLa cells, with IC_50_ values from 9.12 to 12.72 μM. Lung carcinoma A549 cells were much less susceptible to those compounds (IC_50_ values between 21.80 and 92.14 μM). Unfortunately, more sensitive turned out be normal lung cells MRC-5 (IC_50_ values from 18.56 to 81.33 μM). The electron-donating and electron-withdrawing groups of the acetophenone moiety seemed to have no influence on the cytotoxic activity against cancer cells, suggesting that the thiadiazole–chalcone pharmacophore played the crucial role in the cytotoxicity of these compounds. The derivatives most potent in HeLa cells, 5a ((*E*)-*N*-(5-(3,4-Dihydroxyphenyl)-1,3,4-thiadiazol-2-yl)-4-(3-oxo-3-phenylprop-1-en-1-yl)benz-amide), 5c ((*E*)-*N*-(5-(3,4-Dihydroxyphenyl)-1,3,4-thiadiazol-2-yl)-4-(3-oxo-3-(m-tolyl)prop-1-en-1-yl)benz-amide), 5f ((*E*)-*N*-(5-(3,4-Dihydroxyphenyl)-1,3,4-thiadiazol-2-yl)-4-(3-(3-methoxyphenyl)-3-oxoprop-1-en-1-yl)benzamide) and 5 m ((*E*)-*N*-(5-(3,4-Dihydroxyphenyl)-1,3,4-thiadiazol-2-yl)-4-(3-oxo-3-(thiophen-2-yl)prop-1-en-1-yl)benzamide), were subjected to further analyses. Treatment with these compounds caused G2/M cell cycle arrest, triggered caspase-dependent apoptosis and induced DNA damage [88].

Conformational changes in DNA topology, necessary for transcription, replication and recombination of genetic material, are catalyzed by topoisomerases (Topo). Inhibition of the activity of these enzymes reduces DNA synthesis and cell division. Aside from the molecules that inhibit the catalytic activity of Topo, there are also Topo poisons converting this enzyme into a cell poison, leading to irreversible damage of genetic material [89]. Plech and co-workers synthesized 1,3,4-thiadizole derivatives of which two (compound 3 and 4) stabilized the DNA-TopoII cleavable complex, thus acting as topoII poisons. Among them, compound 3, 2-(4-bromophenylamino)-5-(2,4-dichlorophenyl)-1,3,4-thiadiazole, displayed toxicity towards breast cancer MCF-7 and MDA-MB-231 cells (IC_50_ values of 120 and 70 μM, respectively), but did not harm fibroblasts. It diminished DNA synthesis in cancer cells, but not in fibroblasts, and inhibited the activity of topoII (Fig. 2) [90].

### Miscellaneous compounds

Anticancer activity of a series of 5-substituted 2(2,4-dihydroxyphenyl)-1,3,4-thiadiazoles was examined by Matysiak and co-workers [91]. Those compounds displayed cytotoxicity towards human cell lines derived from bladder cancer (HCV29T), non-small lung carcinoma (A549), rectal adenocarcinoma (SW707), and breast cancer (T47D). The most active against HCV29T cells were compound 7, bearing 4-(CH_3_)_3_C–C_6_H_4_ group, and compound 26, bearing 4-CH_3_O–C_6_H_4_–CH_2_O group (IC_50_ values of 3.7 and 1.1 μg/mL, respectively). Cisplatin, used as a reference drug, was much more toxic (IC_50_ of 0.7 μg/mL). Compounds 7 and 26 displayed significant cytotoxicity towards SW707 (IC_50_ values of 4.5 and 5.0 μg/ml, respectively), A549 (IC_50_ values of 12.8 and 7.9 μg/mL, respectively) and T47D (IC_50_ values of 4.0 and 3.0 μg/mL, respectively) cell lines. The activity of cisplatin was comparable against SW707 (IC_50_ value of 4.9 μg/mL), higher against A549 (IC_50_ value of 3.3 μg/mL), but lower against T47D (IC_50_ value of 6.2 μg/mL) cells. The structure–activity relationship (SAR) analysis indicated that aryl derivatives were more active compared to alkyl derivatives, but the influence of the type of aryl ring substituent on the activity was not significant. Joining the aryl ring by means of either –CH_2_– or –OCH_2_– link also did not improve the anticancer activity of compounds [91]. A further detailed quantitative SAR analysis revealed that electron properties of 1,3,4-thiadiazole ring was the most important factor for the activity of these derivatives. The type of substitution at the fifth carbon atom changed charge distribution of this moiety. Molar refractivity (CMR) turned out to be another parameter influencing the activity of 1,3,4-thiadiazole derivatives [92].

In the next study, cytotoxicity of a series of differently substituted in *N*-aryl ring 2-phenyloamino-5-(2,4-dihydroxyphenyl)-1,3,4-thiadiazoles against four aforementioned cell lines was examined. Compound I, with an unsubstituted amine group, showed a weak activity against HCV29T cells with IC_50_ value of 205.7 μM. All *N*-substituted derivatives, except for compounds bearing either 4-CH_3_–3-Cl–C_6_H_3_- or 4-CH_3_O–C_6_H_4_ group, were significantly more potent in this cell line and displayed IC_50_ ranging from 20.7 to 115 μM. Compound VIII with iodine atom in the *para*-position of the *N*-phenyl ring was the most active of the tested derivatives, yet less potent than cisplatin which IC_50_ value was 2.3 μM. This reference drug presented also considerable cytotoxicity in A549, SW707, and T47D cells (IC_50_ values of 11, 16.3 and 20.7 μM, respectively). None of the tested 2-phenyloamino-5-(2,4-dihydroxyphenyl)-1,3,4-thiadiazole derivatives appeared to be more active than cisplatin in either A549 or SW707 cells (IC_50_ values ranging from 17.0 to 98.5 μM and from 19.5 to 96.7 μM, respectively). However, in T47D cell line, most of the tested compounds were more active (IC_50_ values from 9.7 to 19.9 μM) compared to cisplatin (IC_50_ value 20.7 μM). The highest activity displayed derivatives bearing either bromine or iodine atom in the *para*-position of aryl ring as well as 2-methyl-5-chlorophenyl derivative, suggesting an advantageous influence of electron-withdrawing (σ > 0) and hydrophobic (π > 0) substituents and polarizability of bromine and iodine atoms [93].

Among another series of *N*-substituted 2-amino-5-(2,4-dihydroxyphenyl)-1,3,4-thiadiazoles, phenyl derivatives turned out to be relatively active, although their potency was dependent on *N*-aromatic ring-substitution degree. In SW707 cells, the parent compound 4 displayed cytotoxicity comparable to that of cisplatin (IC_50_ values of 4.3 and 4.9 μg/mL, respectively). In three other cell lines, this compound was slightly less active compared to the reference drug. Substitution of phenyl ring with either the lipophilic electron-donating or morpholinoalkyl groups decreased the activity of the parent compound. On the contrary, the compound with the hydrophobic substituents (π > 0) of electron-withdrawing character (α > 0) appeared to be much more promising anticancer agents. Thus, the substitution of the ring with a fluorine atom in the *para*-position resulted in the activity towards HCV29T, SW707, T47D cells (IC_50_ values 6.2, 3.6 and 4.2 μg/mL, respectively) comparable or slightly higher than parent compound 4 and cisplatin. Similarly, the compound with a chlorine atom in the *meta*-position displayed cytotoxicity in HCV29T, SW707 and T47D cells (IC_50_ values 5.4, 3.7 and 3.9 μg/mL, respectively) comparable to that of the parent compound and the reference drug. Either modification did not increase the activity towards A549 cells. However, the substitution of the ring with two chlorine atoms in positions 2 and 4 resulted in the cytotoxicity comparable (IC_50_ value 5.3 μg/mL) in A549 cells or remarkably higher (IC_50_ values of 2.8 and 1.5 μg/mL, respectively) in SW707 and T47D cells to that of compound 4 and cisplatin. This compound turned out to be much less active in HCV29T cells (IC_50_ value 22.8 μg/mL). These results supported the previous notion that 2-amino-1,3,4-thiadiazole acts as pharmacophore of anticancer activity and 2,4-dihydroxyphenyl moiety in position 5 significantly contributes to cytotoxicity. Most likely, such a substituent not only contributes to the favorable hydrophobic-hydrophilic character but also affects electronic properties crucial in compound-target(s) interactions responsible for cytotoxic properties [94].

The promising results obtained for *N*-halogenphenyl derivatives resulted in a further in-depth analysis of those compounds. Among them, 2-(4-chlorophenylamino)-5-(2,4-dihydroxyphenyl)-1,3,4-thiadiazole (herein referred to as CPDT) displayed the ability to diminish the viability of cancer cells. This compound turned out to be most toxic towards human thyroid carcinoma FTC238, colon carcinoma HT-29 and leukemia Jurkat cells (IC_50_ values from 6.4 to 6.7 μM) and mouse teratoma P19 cells (IC_50_ value 8.5 μM). Less susceptible to CPDT treatment were human breast carcinoma T47D, medulloblastoma TE671, astrocytoma MOGGCCM and rat glioma C6 cells (IC_50_ values of 10.7, 15.3, 19.4 and 12.7 μM, respectively). CPDT treatment diminished cancer cell proliferation and migration but did not affect the viability of non-cancerous rat astrocytes, neurons, hepatocytes as well as human fibroblasts [95].

The other derivative, 2-(4-fluorophenyloamino)-5-(2,4-dihydroxyphenyl)-1,3,4-thiadiazole, herein termed FPDT, also diminished the viability of several cancer cell lines. Threshold concentrations of this compound required to elicit cytotoxic effect were as low as 5 μM (human colon cancer HT-29 cells), 10 μM (human lung carcinoma A549 and medulloblastoma TE671 cells), and 25 μM (human neuroblastoma SK-N-AS and rat glioma C6 cells). Moreover, FPDT treatment diminished proliferation and migration of C6 and A549 cells. It should be emphasized that FPDT did not affect non-cancerous cells, as rat astrocytes, neurons and hepatocytes were resistant to this compound up to 100 μM. Furthermore, neurotoxicity caused either by serum-deprivation or glutamate treatment was ameliorated by co-exposure to FPDT, indicating a neuroprotective activity of this compound. Quantum-chemical calculations confirmed that aminothiadiazole moiety acted as pharmacophore of cytotoxic activity of FPDT, and both the 2,4-dihydroxyphenyl and *para*-fluorophenyl substituents intensified its properties [96].

The more detailed molecular analysis revealed that in A549 cells treatment with FPDT decreased phosphorylation level of kinases involved in tumorigenesis, MEK1/2 and ERK1/2, as well as its downstream target, a transcription factor CREB. Furthermore, FPDT treatment enhanced the expression of p27/Kip1, members of the Cip/Kip family of cyclin-dependent kinase inhibitors which block cell cycle progression through the G1/S phase [97]. Indeed, treatment of A549 cells with FPDT increased the number of cells in the G0/G1 phase and decreased the number of cells in the S and G2/M phases [98]. Clearly further studies are required to elucidate whether and to what extend downregulation of the ERK pathway contributes to the anti-cancer properties of FPDT. Of note, our unpublished data strongly indicate that anticancer activity of this compound observed in human glioblastoma cells is causatively linked to downregulation of the AKT pathway, a cascade crucial for the pathogenesis of these tumors. It is, therefore, tempting to speculate that the mechanism underlying FPDT’s activity is related to some upstream signal molecules of the AKT and ERK pathways. For instance, several receptor tyrosine kinases (RTK) have been shown to activate RAS proteins, which in turn modulate both AKT and ERK pathways [99].

Modulation of the other kinases upon treatment with 1,3,4-thiadiazole derivatives was also observed by Cascioferro and co-workers [100]. In this study, a series of 3-(imidazo [2,1-b] [1,3,4]thiadiazol-2-yl)-1*H* indole analogues were synthesized and their anticancer activity was evaluated in pancreatic ductal adenocarcinoma cells. Compounds 12a (3-[6-(Thiophen-3-yl)imidazo[2,1-b] [1,3,4]thiadiazol-2-yl]-1*H*-indole hydrobromide) and 12b (1-Methyl-3-[6-(thiophen-3-yl)imidazo[2,1-b] [1,3,4]thiadiazol-2-yl]-1*H*-indole hydrobromide), exhibited a remarkable antiproliferative activity in SUIT-2, Capan-1 and Panc-1 cell lines with IC_50_ values ranging from 0.85 to 1.70 μM. Moreover, both compounds significantly inhibited the growth and migration of primary patient-derived adenocarcinoma cells and resistant to gemcitabine Panc-1R cells. Treatment with either compound decreased proteolytic activity of matrix metalloproteinases MMP2 and MMP9 and compound 12b diminished the phosphorylation of focal adhesion kinase (FAK), a non-receptor tyrosine kinase which regulates cell proliferation and motility [100].

In another study from the same group, 5-Methoxy-3-[6-(4-nitrophenyl)imidazo[2,1-b][1,3,4]thiadiazol-2-yl]-1*H*-indole, referred to as compound 9c, displayed anticancer activity in the cell lines mentioned above with IC_50_ values of 5.5–5.18 μM. Furthermore, this compound inhibited migration of SUIT-2 and Capan-1 cells more than gemcitabine [101], confirming the previous notion that imidazo[2,1-b][1,3,4]thiadiazole derivatives might be interesting scaffolds for designing new drugs (see “Inhibitors of tubulin polymerization”).

## Conclusions

The anticancer potential of numerous thiadiazole derivatives is undeniable. However, the studies reviewed above have several limitations. First, caution needs to be taken when comparing compounds’ efficacy determined by different assay methods. Second, the activity of the majority of thiadiazole derivatives was evaluated in cancer cells, while their activity in non-cancerous cells remains unknown. Third, in most of the cited studies, the compounds’ efficacy was evaluated in models in vitro. Finally, the molecular targets of the majority of thiadiazole derivatives have not been identified so far. Further in-depth analyses are needed to determine compounds’ selectivity, bioavailability and mode of action. Nevertheless, data depicted above clearly indicate that thiadiazole moiety may be valuable lead structure in the development of new compounds with improved anticancer activity.
